# An Anti-Phospholipase A_2_ Receptor Quantitative Immunoassay and Epitope Analysis in Membranous Nephropathy Reveals Different Antigenic Domains of the Receptor

**DOI:** 10.1371/journal.pone.0061669

**Published:** 2013-04-29

**Authors:** Astrid Behnert, Marvin J. Fritzler, Beina Teng, Meifeng Zhang, Frank Bollig, Hermann Haller, Andrej Skoberne, Michael Mahler, Mario Schiffer

**Affiliations:** 1 Division of Nephrology, Hannover Medical School, Hannover, Germany; 2 Faculty of Medicine, University of Calgary, Alberta, Canada; 3 INOVA Diagnostics, INC., San Diego, California, United States of America; Duke University Medical Center, United States of America

## Abstract

The phospholipase A_2_ receptor (PLA_2_R) was recently discovered as a target autoantigen in patients with idiopathic membranous nephropathy (IMN). Published evidence suggests that the autoantibodies directed towards a conformation dependent epitope are currently effectively detected by a cell based assay (CBA) utilizing indirect immunofluorescence (IIF) on tissue culture cells transfected with the PLA_2_R cDNA. Limitations of such IIF-CBA assays include observer dependent subjective evaluation of semi-quantitative test results and the protocols are not amenable to high throughput diagnostic testing. We developed a quantitative, observer independent, high throughput capture immunoassay for detecting PLA_2_R autoantibodies on an addressable laser bead immunoassay (ALBIA) platform. Since reactive domains of PLA_2_R (i.e. epitopes) could be used to improve diagnostic tests by using small peptides in various high throughput diagnostic platforms, we identified PLA_2_R epitopes that bound autoantibodies of IMN patients. These studies confirmed that inter-molecular epitope spreading occurs in IMN but use of the cognate synthetic peptides in immunoassays was unable to conclusively distinguish between IMN patients and normal controls. However, combinations of these peptides were able to effectively absorb anti-PLA_2_R reactivity in IIF-CBA and an immunoassay that employed a lysate derived from HEK cells tranfected with and overexpressing PLA_2_R. While we provide evidence of intermolecular epitope spreading, our data indicates that in addition to conformational epitopes, human anti-PLA_2_R reactivity in a commercially available CBA and an addressable laser bead immunoassay is significantly absorbed by peptides representing epitopes of PLA_2_R.

## Introduction

Membranous nephropathy (MN) is one of the most common causes of idiopathic nephrotic syndrome in adults [1], [2]. Patients typically present with nephrotic range proteinuria, edema, hypoalbuminemia and hyperlipidemia. Two different forms of MN have been identified: a primary of idiopathic form (IMN), which is found in 80% of MN patients, and a secondary form associated with various malignancies, autoimmune diseases and some infections [1], [2]. Certain histopathological features that can help distinguish IMN from secondary MN include the presence of immune complex deposits in the mesangium and subendothelial space in secondary MN as compared to the exclusively subepithelial and intramembranous deposits seen in IMN. IgG_4_ has been reported to be the predominant Ig subclass in IMN, whereas IgG_3_ and IgG_2_ tend to be more dominant in glomerular deposits of secondary MN [3].

In the 1950s, Heymann and his associates developed an experimental animal model wherein rats developed severe proteinuria after active or passive immunization with certain antigenic fractions of proximal tubular brush border [4]. The histopathological features of renal involvement in this model were similar to those seen in human IMN and additional studies showed that antibodies bound to a membrane receptor expressed on rat renal podocytes, which was eventually identified as megalin [5]. Nevertheless, there have been limitations to directly relating observations in this animal model to human disease. First, to date there is no evidence indicating that megalin is expressed on human podocytes. Furthermore, in contrast to anti-megalin antibodies being capable of activating the complement pathway that leads to podocyte damage, antibodies of the IgG_4_ subclass that are characteristic of human MN, are believed to be ineffective activators of the classical pathway of complement [6], [7].

Evidence that *in situ* formation of immune complexes is responsible for human MN was first described in a single case report in 2004 by Debiec et al [8]. In that report, anti-neutral endopeptidase (NEP) antibodies of a NEP deficient mother crossed the placenta into the fetal circulation where they bound the glomerular basement membrane as well as NEP on fetal podocytes. The disease process in this fetus was reminiscent of the Heymann nephritis model in rats [8], [9].

More recently in 2009, Beck et al described the M-type phospholipase A_2_ receptor (PLA_2_R) as an autoantigen in MN based on immunoblot analysis and mass spectrometry [10]. PLA_2_R is a 180 kDa type I transmembrane protein that belongs to the C-type animal lectin family such as the mannose receptor [11]. PLA_2_R is composed of a large extracellular region consisting of a N-terminal cysteine-rich region (C-R), a fibronectin type II domain (FNII), eight C-type lectin like domains (CTLD), and a short intracellular C-terminal region. Although PLA_2_R is expressed on alveolar type II epithelial cells and on neutrophils, data to date suggest that it is mainly restricted to kidney podocytes [5]. PLA_2_R has been found to promote senescence in human fibroblasts and is involved in both positive and negative regulation of secretory PLA_2_.

Autoantibodies directed to PLA_2_R were found in 52–82% of individuals with IMN [10] but only to a small percentage (5–25%) in sera from patients with secondary MN as detected by Western immnunoblot, IIF-CBA and ELISA [12]–[14]. The observation that some patients with IMN do not have the PLA_2_R autoantibodies could be explained by limitations of current immunoassays, and/or the absence of these autoantibodies during treatment or inactive disease. In addition, autoantibodies to PLA_2_R may not be a universal feature of IMN because other autoantibodies, such as those directed against α-enolase or aldose reductase, have previously been reported in patients with IMN, albeit at a much lower frequency [15], [16]. Proteinuria as a traditional marker of disease activity in IMN correlates with, but does not perfectly parallel, anti-PLA_2_R levels [17]. Proteinuria often remains elevated when antibody levels are undetectable, which has lead to the hypothesis that there might be other yet to be identified serum autoantibodies in IMN. Since anti-PLA_2_R are strongly associated with disease activity, it is thought they are pathogenic but no clear mechanisms of pathogenesis for IMN have been identified so far [18], [19].

In order to detect and quantify circulating anti-PLA_2_R antibodies, an indirect immunofluorescence cell based assay (IIF-CBA) [20], [21] and ELISAs have been developed. For the most part, ELISAs based on recombinant human PLA_2_R c-DNAs expressed in a human cell lines have been developed in individual labs and are not widely validated [12], [22]. The commercially available IIF-CBA diagnostic kit contains a mosaic of two biochips in each well: one overlaid with human embryonic kidney (HEK)293 cells transfected with and over-expressing the PLA_2_R cDNA, while the "control" biochip contains non-transfected HEK cells. The non-transfected cells are important in assessing positive reactions because human autoimmune sera often have a variety of autoantibodies directed against nuclear, cytoplasmic and cell surface targets, making interpretation of results quite challenging even for an experienced technologist. While the IIF-CBA is relatively inexpensive and easy to perform, it has some limitations in that it is not amenable to high throughput diagnostics used in many larger diagnostic laboratories; it is semi-quantitative; interpretation of the IIF result is largely subjective and it is relatively labour intensive.

Therefore, our goal was to develop a quantitative, high throughput immunoassay that has at least comparable sensitivity and specificity of the IIF-CBA and avoids the subjective assessment of IIF especially in the presence of other autoantibodies that may obscure a true positive result. We anticipated that such a new quantitative diagnostic assay might be more representative of the immunological state of the disease, a feature which in turn might benefit patients. To this end, we tested recombinant over-expressed PLA_2_R as a potential target on an addressable laser bead immunoassay (ALBIA) diagnostic platforms and examined overlapping PLA_2_R 15mer peptides representing the full length protein as an approach to identifying a specific epitope which could then be adapted to an improved immunoassay by using smaller peptides [23].

## Materials and Methods

### Patients and Controls

Patient and control serum samples were collected at the Medical School Hannover, (Germany). This study was approved by Ethics Committee of Medical School Hannover, Germany, (Nr: 1246–2011) and patient data were anonymously used under consideration of the latest version of the Helsinki Declaration of human research ethics.

### Indirect Immunofluorescence Cell Based Assay for anti-PLA_2_R

Patient and control serum samples were identified as PLA_2_R positive or negative samples based on their reactivity in a commercially available indirect immunofluorescence cell based assay (IIF-CBA: Euroimmun, Luebeck, Germany) performed according to the manufacturer's protocol.

### PLA_2_R Construct, Western Blot and Indirect Immunofluorescence

A PLA_2_R isoform1 (Accession: Q13018, 1463 aa, 180 kDa) was constructed and inserted into a GFP vector (Clontech Laboratories Inc., Saint-German-en-Laye, France). In order to test reactivity of our construct, we performed IIF with patient serum as primary antibody diluted 1∶100 in phosphate buffered saline (PBS) and FITC-conjugated mouse anti-human IgG (Santa Cruz Biotechnology Inc., Santa Cruz, CA, USA) diluted 1∶1000 in PBS as secondary antibody. Western immunoblots were performed on transfected cell lysates (described below) with a commercial goat anti-PLA_2_R (Acris Antibodies, Herford, Germany), mouse anti-GFP (Santa Cruz) and patient sera as described above.

### Protein Coupling, Blocking and Storage onto Microspheres

Details of the procedure and components of the coupling procedures and assay development are found in Methods S1. Briefly, 10 mg of 1-ethyl-3-(3-dimethylaminopropyl)carbodiimide (EDC) and normal human serum (NHS) were dissolved in 200 *µ*l of Activation Buffer. A desired volume of beads (Luminex Corp., Austin, TX, USA) was pipetted into micro tubes (USA Scientific Inc. Ocala, FL, USA) and centrifuged at 14,000 rpm for 1 min. The supernatant was carefully decanted, the desired amount of activation buffer was added and the beads were resuspended by gentle sonication and vortexing. Diluted EDC and NHS were added and the beads sonicated and vortexed again followed by a 20 minute-incubation in the dark at room temperature. While the beads were incubating, protein samples were diluted to the optimal concentration in Coupling Buffer (usually 50 *µ*g/ml; see Methods S1). After incubation, beads were centrifuged at 14,000 rpm for 3 minutes and the supernatant decanted before adding coupling buffer at 2–3 times the original bead volume. The microspheres were again sonicated and vortexed before centrifugation at 14,000 rpm for another 3 minutes. The supernatant was decanted and protein was coupled to microspheres by adding the optimal amount of protein to the microspheres, which were resuspended as described above. The beads were then incubated overnight at 4°C on rotator and then stored at 4°C in the dark until required for use.

### ALBIA Utilizing PLA_2_R Peptides

2 µl of suspended beads in solution (Luminex Corp.), 35 µl of horse radish experiment (HRP) sample diluent (INOVA Diagnostics Inc.) and 5 µl of diluted serum were pipetted into the wells of light tight microtitre plates (Luminex Corp.). The plate was covered so as to avoid sustained exposure of the beads to ambient light and incubated at 4°C on a shaker set at 600 rpm over night. The next day, 40 µl of diluted, phycoerythrin (PE) conjugated secondary antibody (goat anti-human IgG/mouse anti-human IgG_4_, 1∶50/1∶25 in HRP sample diluents, Jackson ImmunoResearch, West Grove, PA, USA) were added and incubated with agitation at 600 rpm for 30 min at room temperature in the dark. Plates were analyzed by ALBIA using a Luminex-100 flow apparatus.

### Cell Lysates

HEK293 cells (American Type Culture Collection, Cedarlane, Burlington, ON, Canada) were seeded in culture plates (NuncUpCell Surface 10 cm, Thermo Fisher Scientific, Langenselbold, Germany) and incubated for one day to enhance attachment before transfection with the PLA_2_R construct as described above. It was determined that at 48 hours after transfection, HEK293 cells most efficiently over-expressed PLA_2_R as determined by IIF. Hence, subsequent cell lysates were prepared by first washing cells with cold PBS and then harvesting cells on ice using cold NETN buffer (150 mM NaCl, 1 mM EDTA, 50 mM Tris-HCl (pH 7,4), 1% Nonidet P-40/Tergitol, protease inhibitor (Complete Mini, Roche, Indianapolis, IN, USA), phosphatase inhibitor (PhosSTOP, Roche, Indianapolis, IN, USA). Lysates were stored at −80°C overnight and then centrifuged for 15 min at 11.000 rpm at 4°C. The supernatant was transferred into a new tube and stored at −20°C.

### ALBIA Utilizing Cell Lysates

A 200 µl suspension of ALBIA beads (MicroPlex Microsphere (non-magnetic) LC10052, MiriaBio Group, San Francisco, CA, USA) coupled with 50 µg/ml mouse anti-GFP (Abcam, Toronto, ON, Canada) were added to 1 ml of PLA_2_R transfected HEK293 cell lysates and incubated on a shaker for 1 hour at room temperature. Beads were then washed twice with 500 µl Wash Buffer (Millipore Corp., Billerica, MA, USA) and once with 500 µl Blocking/Storage Buffer (PBS, 0.1% bovine serum albumin (BSA), 0.02% Tween-20, 0.05% azide, pH 7.4) before resuspending the beads in 200 µl Blocking/Storage Buffer. 2 µl of the resuspended beads, 30 µl of HRP sample diluent (INOVA Diagnostics Inc., San Diego, CA, USA) and 10 µl of diluted serum (1∶100 in HRP sample diluent) were pipetted into the wells of microtiter plate, covered and incubated on a shaker, 600 rpm, for 1 hour at room temperature. 40 µl of PE conjugated goat anti-human IgG (1∶50/HRP sample diluent, Jackson ImmunoResearch) or 40 µl of PE conjugated mouse anti-human IgG_4_ (1∶25 in HRP sample diluents, Jackson ImmunoResearch) was then added and the plate was incubated for an additional 30 min at room temperature. The reactivity of individual sera was then analyzed using a Luminex-100 plate reader.

### Epitope Mapping

For epitope mapping, sequential human PLA_2_R isoform1 (Accession: Q13018) peptides were synthesised on a cellulose membrane using SPOT technology as previously described [24]. Briefly, peptides of 15 amino acids (aa) overlapping by 5 aa were produced by delivering activated amino acids to the corresponding spot on a derivatized cellulose membrane. In between cycles, aa were fluorenylmethoxycarbonyl (Fmoc) deprotected, so that coupling of the next aa was possible. Spots were run in duplicate to gain more reproducible results.

Peptide-antibody interactions were observed by first blocking the membrane with 3% milk in Tris buffered saline (TBS) for 1 hour at room temperature, then incubating with diluted serum (1∶00 in 3% milk/TBS) for 2 hours. Anti-PLA_2_R antibodies were detected by incubating the membrane with anti-human IgG HRP conjugated (1∶10.000/TBS, Jackson ImmunoResearch) for 1 hour and then developed for electrochemiluminescence (ECL) (Amersham™ ECL™ Western Blotting Detection Reagents by GE Healthcare, Piscataway, NJ, USA).

It is possible to reprobe stripped and regenerated membranes for several cycles To accomplish this, the membrane was immersed in a solution containing 100 mM β- mercaptoethanol, 2% sodium dodecyl sulfate (SDS); 62,5 mM Tris-Cl pH 6,7 and it was incubated for 30 minutes at 50°C. Thereafter the membrane is washed in several changes of TBS and to verify successful stripping, the membrane was incubated with the secondary antibody only and checked with ECL.

Randomly selected sera from seven patients with MGN that were positive for anti-PLA_2_R antibodies, as well three MGN anti-PLA2R negative sera and five normal healthy controls were tested on the membranes. Commercially available rabbit anti-PLA_2_R antibody (1∶500/TBS, Abcam, rabbit polyclonal anti-PLA_2_R, immunogen: synthetic peptide derived from the C-terminal domain of human PLA_2_R) served as a positive control. Donkey anti-human IgG HRP conjugated (1∶10.000/TBS, Jackson ImmunoResearch), mouse anti-human IgG_4_ HRP conjugated (1∶1.000/TBS, Abcam) or HRP conjugated goat anti-rabbit IgG (1∶5.000/TBS, Jackson ImmunoResearch) were utilized as secondary antibodies when appropriate.

### PLA_2_R Peptide ELISA

100 µl of 2 µg/µl peptides diluted in coating buffer representing the PLA_2_R reactive epitopes were commercially produced (EZ Biolab, Carmel, IN, USA) and coated onto 96-well microtiter plates. The covered plate was then incubated overnight at 4°C and then washed twice by filling each well with 200 µl of PBS. The plate was then blocked by incubating 200 µl of blocking solution (5% skim milk in PBS) per well overnight at 4°C. After washing the plate twice, 100 µl of diluted primary antibody (patient serum diluted 1∶100 in PBS) were pipetted into each well and the covered plate was incubated for 2 hours at room temperature. After the primary antibody was removed and the plate was washed four times with PBS, the plate was incubated with diluted secondary antibody (1∶10.000/PBS, HRP conjugated mouse anti-human IgG, Jackson ImmunoResearch; 1∶5.000/PBS, or HRP conjugated anti-human IgG_4_, Abcam) for 1–2 hours at room temperature. The plates were washed again in four changes of PBS to remove unbound proteins. 100 µl of substrate solution was added to each well followed by 100 µl of stop reagent. The absorbance at 450 nm (reference: 540 nm) was read on a JANUS® MDT Automated Workstation (PerkinElmer Inc., Waltham, MA, USA).

### Absorption Assay

Individual and mixtures of all peptides produced as described above (Table 1) were stored at 5 mg/ml at −80°C. The peptides were added to achieve 14, 70, and 126 µg in each absorption reaction. Controls included an equivalent amount of peptide buffer (1% BSA in PBS) containing no competing peptides and an unrelated synthetic peptide GE-1 (RCD8/Hedls; a 22mer peptide representing a major epitope commercially prepared by Pepceutiucals Ltd., Nottingham, United Kingdom) [25] as a negative reference control used at the same concentrations as the PLA_2_R peptides. Anti-PLA_2_R sera were separately tested to achieve the usual working dilution of 1∶100. After incubation overnight at 4°C, the various solutions were assayed as per normal protocols in the commercial IIF-CBA and in the ALBIA (see ALBIA utilizing cell lysates and IIF-CBA above for details).

**Table 1:** Amino Acid Sequences of Consensus PLA_2_R Epitopes Identified by SPOT.

#	Amino acid sequence	Amino acid position	Location
1	LLLGAPRGCAEGVAAALTPE	16–25	C-R
2	LSWSEAHSSCQMQGGTLLSI	256–265	CTLD 1
3	EALRSCQADNSALIDITSLA	401–420	CTLD 2
4	HAQHFCAEEGGTLVA	986–1000	CTLD 6
5	GYGFVCEKMQDTSGH	1091–1105	CTLD 6
6	MSFEAAHEFCKKEGS	1271–1285	CTLD 8
7	EGLWQLSPCQEKKGFICKMEADIHT	1361–1385	CTLD 8

## Results

The full-length human PLA_2_R construct that was cloned into a green fluorescent protein (GFP) vector and over-expressed in HEK293 cells was verified by standard sequencing techniques. Successful transfection and reactivity of the GFP construct was confirmed by Western immunoblot employing commercially available anti-GFP and anti-PLA_2_R antibodies as well as patient samples on transfected HEK293 cell lysates (Figure S1).

**Figure 1:**
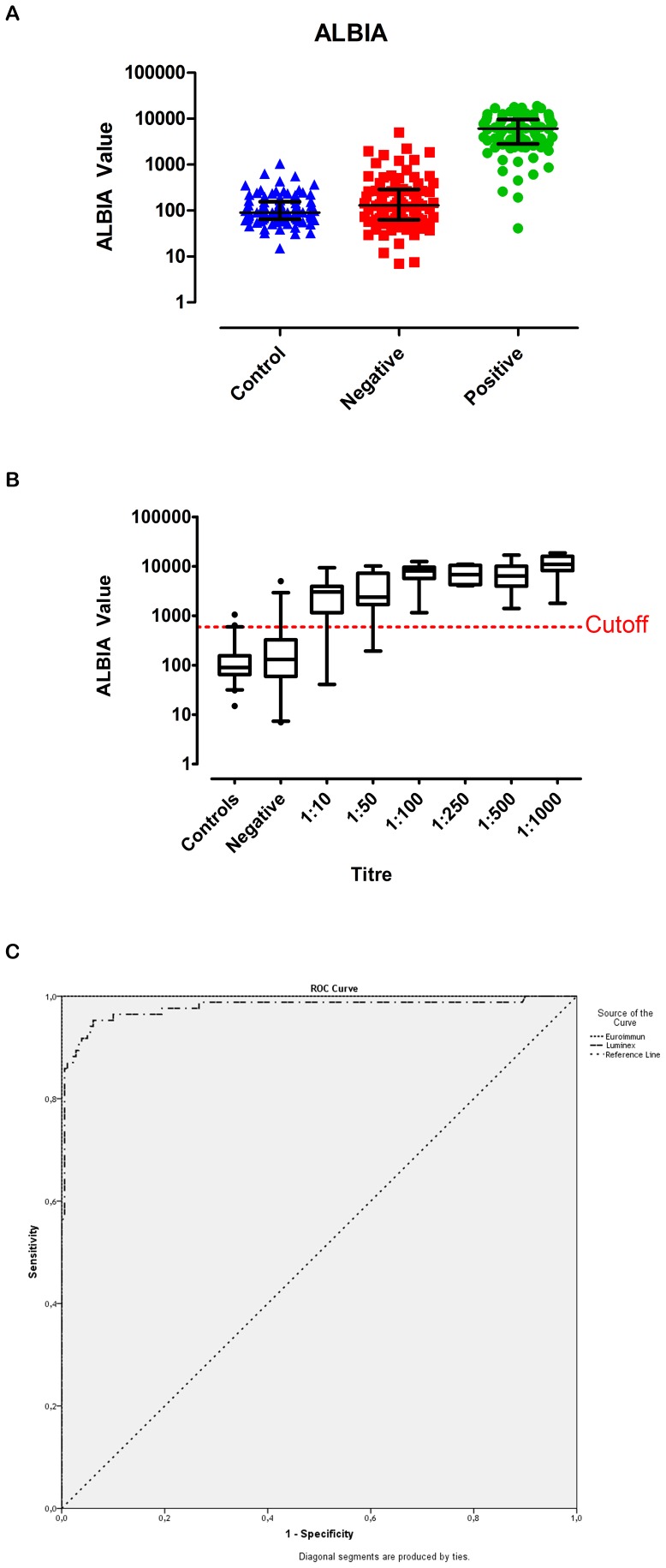
ALBIA (Luminex) IgG. *Panel A:* The median of ALBIA fluorescence units of anti-PLA_2_R positive IMN samples were compared to IMN samples negative for anti-PLA_2_R antibodies and control samples (normal healthy controls and unrelated inflammatory disease controls).Fluorescence values of anti-PLA_2_R positive samples were significantly higher than values of anti-PLA_2_R negative samples and of controls (p-values<0.0001). [Median with interquartile range]. *Panel B:* ALBIA readings were analyzed according to antibody titres determined on CB-IIF. Samples with a high titre on CB-IIF had often a high fluorescent value on the bead-based assay but ALBIA readings did not correlate with CB-IIF titres. [Whiskers: 2.5–97.5 percentile]. *Panel C:* A ROC curve is a graphical plot illustrating the performance of a binary classifier system and is used to evaluate diagnostic tests. Sensitivity (fraction of true positives out of positives) is plotted versus 1-specificity (the fraction of false positives out of negatives). Here, our established ALBIA using HEK cell lysates is compared to the EUROIMMUN IIF-CBA. The EUROIMMUNIIF-CBA is a commercially available immunoassay for anti-PLA_2_R and therefore defined the outcome (anti-PLA_2_R positive vs. anti-PLA_2_R negative).Thus, the IIF-CBA perfectly classifies patients with high sensitivity and specificity. With an area under the curve (AUC) of 0.978, the ALBIA is very close to the CB-IIF assay.

Next, when ALBIA beads were indirectly coupled with the full length protein captured from cell lysates and tested with sera from 165 patients with membranous nephropathy; 85 (52%) of those tested positive, whereas 80 (48%) tested negative for anti-PLA_2_R antibodies on CBA. As controls, sera from 50 normal healthy controls, 25 systemic lupus erythematosus (SLE) patients and 25 patients with granulomatosis with polyangiitis (GPA; formerly Wegener’s granulomatosis) were also tested on this new ALBIA. As individual groups of sera, the fluorescence median value of IIF-CBA positive serum samples in the ALBIA was significantly higher than values observed with IIF-CBA negative, as well as controls. ROC analysis was performed to compare the ALBIA to the IIF-CBA assay because the latter was regarded the only reliable commercially available immunoassay and was hence was used as the reference standard to define the outcome (anti-PLA_2_R positive vs. anti-PLA_2_R negative). Thus, the area under the curve (AUC) for the IIF-CBA assay was set at 100% (Figure 1; Figure S2). By comparison, the ALBIA curve covered 97.8% and with the cut-off value calculated from the ROC analysis, the assay classified patients with a sensitivity of 95.3% and a specificity of 93.9%. The ALBIA was also performed using anti-human IgG_4_as secondary antibody leading to similar results with less background and a lower cut-off value (AUC: 96.3%; sensitivity: 94.1%; specificity: 96.1%). In order to exclude nonspecific antibody binding to GFP moieties, samples were also tested on beads coupled with moc-GFP transfected HEK cell lysates and to an unrelated GFP-coupled aquaporin 4 protein; both of which showed ALBIA fluorescence values well below cut-off values (data not shown).

**Figure 2:**
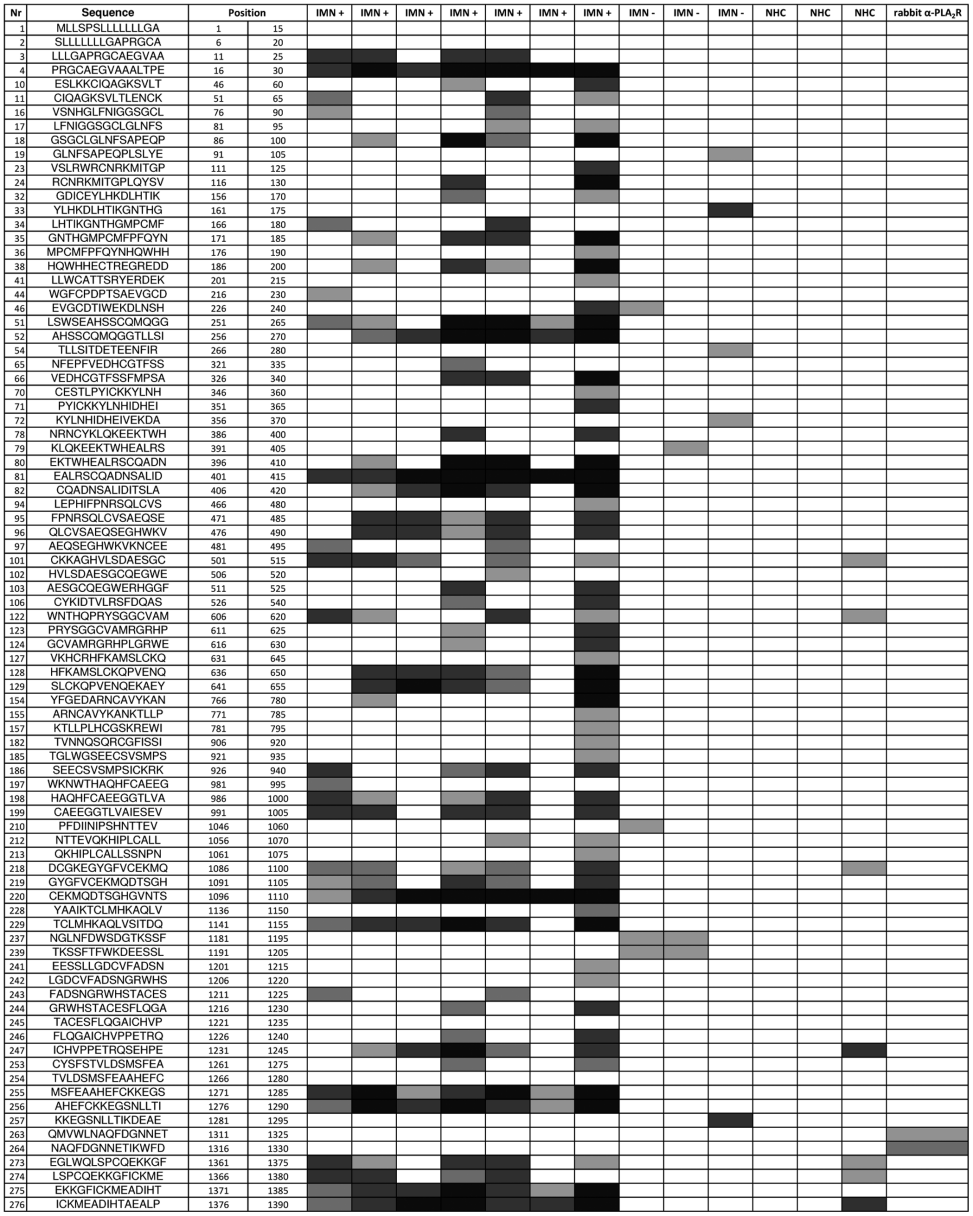
Epitope Mapping of PLA_2_R Peptides with anti-human IgG_4_. Grey scale heat map representation of results from SPOT used to detect PLA_2_R epitopes. Peptide membranes were probed with 10 randomly selected IMN samples that had previously been tested by IIF-CBA for anti-PLA_2_R antibodies (7 positive (IMN+) and 3 negative (IMN-)), as well as 5 normal healthy controls (NHC). The positive control rabbit antibody to PLA_2_R reacted with the expected peptide used as the immunogen. Consensus epitopes and their respective PLA_2_R domains derived from this analysis are illustrated in Figure 3 and summarized in Table 1.

**Figure 3:**
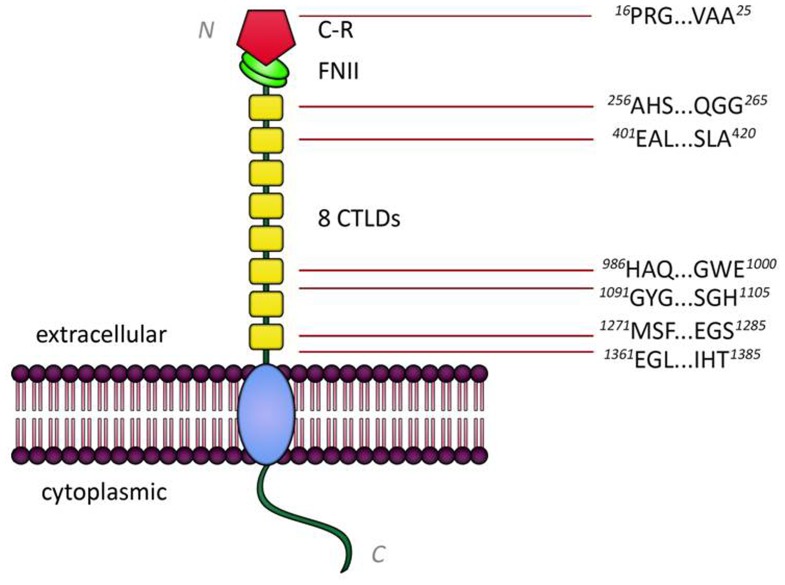
PLA_2_R schematic plot of the seven potential antigen determinants identified by epitope mapping. All of the determinants identified by epitope mapping were located in the extracellular domain of PLA_2_R and are ∼10 to 25 aa long. Only one epitope is not in the C-type lectin like domains of the receptor. *[C-R,cysteine-rich region; FNII, fibronectin type II domain; CTLDs, C-type lectin like domains; N, N-terminal end; C, C-terminal end].*

**Figure 4:**
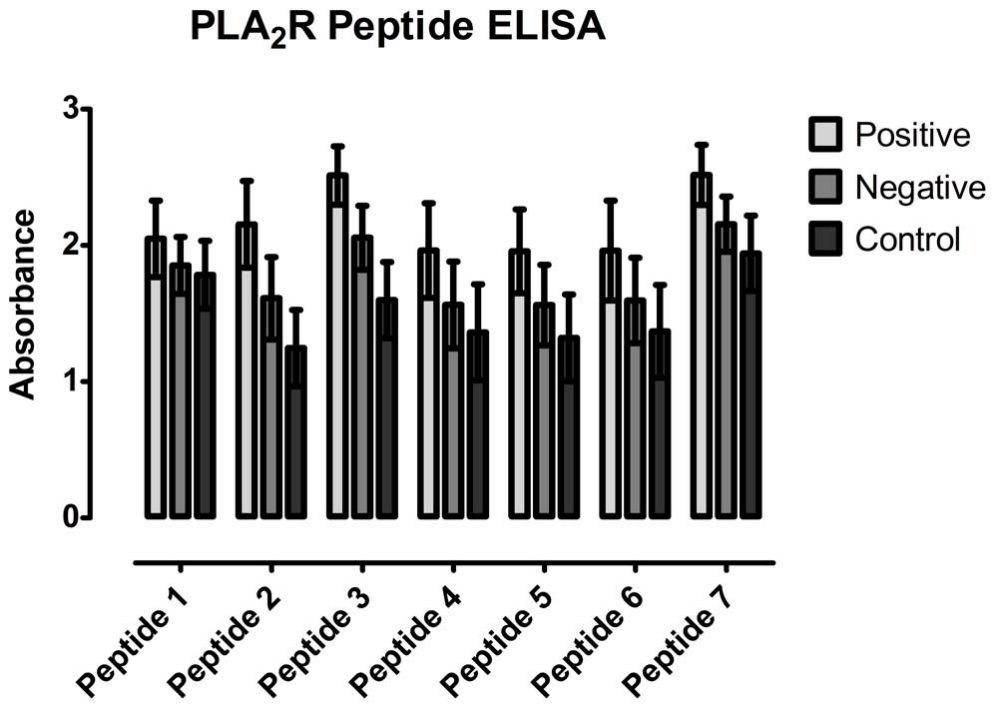
ELISA of synthesized PLA_2_R peptides. To verify potential epitopes, synthetic peptides (see Table 1) were tested by ELISA. Absorbance of patient samples tested positive on the CB-IIF assay was higher than of patient samples tested negative and normal healthy control samples but the difference was not statistically significant (p>0.05).

Assuming that further knowledge about antibody-antigen interactions could improve assay development, we proceeded to determine epitope(s) bound by the human anti-PLA_2_R autoantibodies by using SPOT technology [24]. Overlapping 15mer peptides representing the full length PLA_2_R protein were synthesized on nitrocellulose membranes and potential epitopes detected by conventional Western blot techniques. First, HRP conjugated anti-human IgG (polyspecific) was used as a secondary antibody but this produced a high background signals. Since it was reported that autoantibodies in IMN predominantly belong to the IgG_4_ subclass, HRP conjugated anti-human IgG_4_ was consequently used as a secondary antibody (Figure 2). This was attended by much lower background and stronger signals (Figure S3; Table S1) localized to seven consensus epitopes; all of which were located in the extracellular domain of PLA_2_R (Figure 3). The antigen determinants identified by epitope mapping encompassing 10 to 25 aa were localized to the C-type lectin like domains (CTLDs) of the receptor except for one that was localized to the N-terminal cysteine-rich region (C-R). Of the epitopes located in the CTLDs of the receptor, one was located in CTLD1, one in CTLD2, two were located in CTLD6 and the last two were found to be in CTLD8 (Table 1). Protein Basic Local Alignment Search Tool (BLAST NCBI) search and a cut-off of >80% sequence similarity, determined that these peptides did not share sequence similarity to or alignment with other proteins. Since no strong homology was found using BLAST, molecular mimicry is unlikely and thus further supports the specificity of the peptides. In order to verify the reactivity of the identified epitopes in a diagnostic immunoassay, synthetic peptides were tested by ELISA and ALBIA. Although the absorbance or fluorescence units of positive samples were higher than negative or control samples, these differences were not significant (Figure 4; Figure S4).

**Figure 5:**
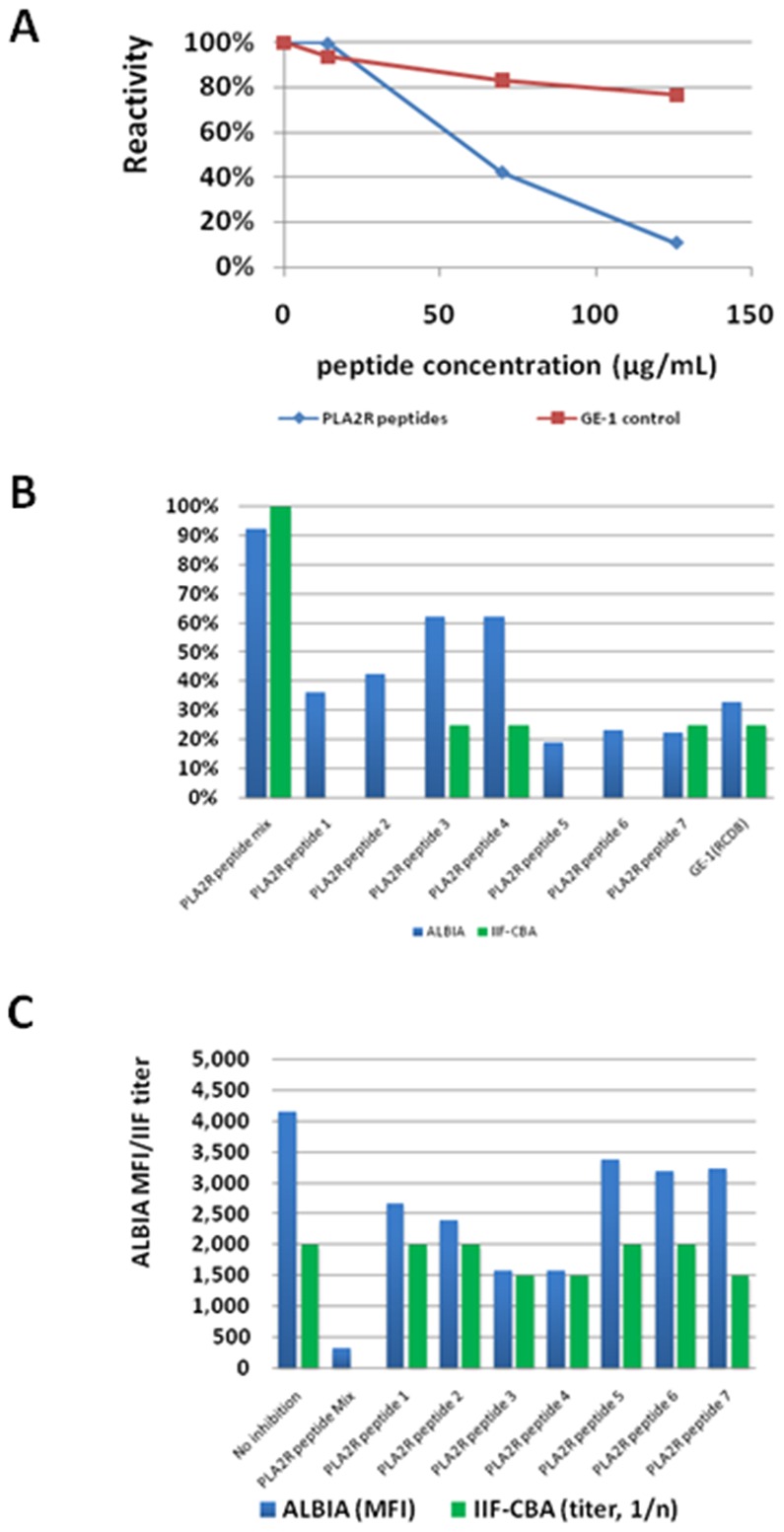
Illustrative Inhibition studies of anti-PLA_2_R antibodies using synthetic peptides with an anti-PLA_2_R positive serum sample. *Panel A:* Different concentrations of a mixture of PLA_2_R derived peptides were used to inhibit the reactivity to the PLA_2_R whole molecule in an addressable laser bead assay. The reactivity showed a significant, dose dependent inhibition. The inhibition with a control peptide (GE-1) was significantly lower. All values are expressed as residual reactivity after inhibition (in %) compared to the sample without inhibitor or control. *Panel B:* The peptide mixture together with seven individual PLA_2_R peptides and a control peptide were used at a concentration of 126 µg/mL. Besides the peptide mixture, peptide 3 and peptide 4 showed inhibition of anti-PLA_2_R antibodies. All values are expressed as residual reactivity after inhibition (in %) compared to the sample without inhibitor or control. *Panel C:* The peptide mixture together with seven individual PLA_2_R peptides and a control peptide were used at a concentration of 126 µg/mL. Besides the peptide mixture, peptide 3 and peptide 4 showed inhibition of anti-PLA_2_R antibodies. All values are expressed as ALBIA median fluorescence intensities (MFI) or titer by indirect immunofluorescence on cell-based assay (IIF-CBA).

In order to determine if the identified peptides could inhibit anti-PLA_2_R binding to the full length chimeric PLA_2_R, a peptide absorption assay was performed on anti-PLA_2_R positive samples from two different patients using both the IIF-CBA and ALBIA. A mixture of the PLA_2_R peptide epitopes identified by SPOT, decreased, in a dose dependent manner, the reactivity to the full length recombinant molecule by approximately 90% in both the ALBIA and IIF-CBA (Figure 5). By contrast, at equivalent concentrations the GE-1 (RCD8) peptides were significantly less efficient in absorbing anti-PLA2R reactivity. In addition to the peptide mixture, peptides 3 and 4 showed intermediate inhibition of anti-PLA_2_R antibody reactivity.

## Discussion

We have developed a new ALBIA using cell lysates that bear the full-length recombinant human protein that reliably detects anti-PLA_2_R antibodies in human IMN sera. Currently, a commercially available immunoassay for determining anti-PLA_2_R antibodies is a semi-quantitative IIF-CBA but it is not well suited to high throughput laboratory platforms and can be troubled by subjective interpretation.

Previous studies by Beck et al [10] found that the majority of autoantibodies in IMN patients bind to a conformational (i.e. discontinuous) epitope. Nevertheless, we were interested in using SPOT technology to explore the possibility of inter-molecular epitope spreading and also hoped to identify a peptide domain that was major antigenic determinant on PLA_2_R isoform1. SPOT technology is a powerful tool that can be used to screen hundreds to thousands of peptides for antibody binding and has been used successfully to identify linear epitopes with clinical relevance [26]–[28]. Our SPOT data clearly indicated that the PLA_2_R autoantibodies are directed against several epitopes, although, we were unable to confirm a previously reported epitope [3]. Unfortunately, despite strong reactivity on cellulose membrane matrices, other immunoassays (i.e. ELISA) were unable to conclusively distinguish between patients and normal controls, even when the synthetic PLA_2_R peptides were tested individually or in various combinations (data not shown). One possible explanation for this apparent paradox is that smaller peptides bound to solid phase matrices may limit autoantibody binding by stearic hindrance.

Even though the synthetic peptides representing potential PLA_2_R epitopes were not effective as analytes in our ELISA or ALBIA platforms, the absorption experiments demonstrated that a mixture of all peptides and to a lesser extent peptides 3 and 4, were able to inhibit binding of anti-PLA_2_R to the full length chimeric protein employed in our ALBIA and to PLA_2_R overexpressed in the IIF-CBA. The relative ineffectiveness of single peptides (representing one single epitope) in completely absorbing all anti-PLA_2_R reactivity was expected because our data clearly showed that the anti-PLA_2_R responses encompasses more than a single epitope. Nevertheless, marginal effectiveness of single peptides observed for peptides 3 and 4 is of interest and merits further study. It is important to appreciate that the same dynamics cannot be expected for every anti-PLA_2_R serum because of the possibility of any number of combinations or permutations of epitopes that would be targeted. It is important to point out that reactivity in the IIF-CBA, which is thought to primarily represent a conformation-dependent immunoassay, was also significantly absorbed by the peptide mixture indicating that even in this assay the epitopes available for antibody binding are quite diverse. In addition to the ALBIA and IIF-CBA, we are anxious to collaborate and determine if similar effects are observed in ELISAs that have been developed [12], [22].

Our observations that anti-PLA_2_R antibodies recognize shorter peptide domains and also absorb anti-PLA_2_R reactivity but fail to bind to the full-length protein in immunoblot experiments is consistent with previous findings in other autoantibody systems. For example, some autoepitopes represent cryptic epitopes that become accessible only after certain conformational changes, a feature demonstrated for beta 2 glycoprotein 1 [29]–[31]. Whether the identified PLA_2_R epitopes contribute to the pathophysiology of IMN remains speculative and requires further research. In addition, it is often assumed that short peptides do not bear conformational epitopes, a concept that does not take into consideration that protein folding and conformation (i.e. helices) can be present as part of natural physicochemical interactions of the constituent amino acids thereby providing continuous conformational epitopes. In addition, it is important to appreciate that even when proteins are “denatured” using various chemical modalities (i.e. mercaptoethanol, dithiothreitol, or SDS) that transfer from SDS polyacrylamide gels to solid phase matrices through an alcohol interphase can result in significant protein refolding. We attempted to chemically reduce our full length chimeric PLA_2_R to expose potentially hidden epitopes and thereby demonstrate enhanced reactivity, but this became technically challenging as the proteins were precipitated using this approach (data not shown).

As noted above, instead of solely targeting a particular conformational PLA_2_R epitope as reported by Beck et al [10], human autoantibodies to PLA_2_R appear to target multiple domains of the receptor, a finding that supports the phenomenon of inter-molecular epitope spreading of B cell responses [32]–[34]. Epitope spreading, defined as the diversification of epitope specificity from its initial focus, has already been described in other autoimmune diseases such as SLE, multiple sclerosis, type-1 diabetes and myasthenia gravis [34] and is suggested to result from tissue damage when so called cryptic (hidden or sequestered) epitopes are exposed. However, it is also considered that epitope spreading might play a protective role, e.g. to enhance efficiency of tumor clearance by up-regulating immune responses or, in contrast, to down-regulate immune responses in autoimmunity [34]. Since we are the first to describe multiple PLA_2_R epitopes that bind to IMN sera as evidence to support the concept of epitope spreading, further studies (i.e. analysis of sequential sera) are necessary to validate and examine the wider clinical relevance of these observations.

In summary, we developed a new immunoassay (ALBIA) for detecting PLA_2_R autoantibodies in IMN sera. We provide evidence that binding of autoantibodies to PLA_2_R domains is likely more complex than previously thought. Some of our findings supporting earlier reports that human IMN autoantibody binding to PLA_2_R depends on conformational epitopes. However, we identified multiple different epitopes on PLA_2_R that bind autoantibodies of IMN patients, a feature that supports a PLA_2_R driven B cell response and B cell production involves autoantibodies that demonstrate inter-molecular epitope spreading in IMN. Future studies in larger patient cohorts are necessary to validate these findings and to analyze if inter-molecular epitope spreading and autoantibody reactivity to specific regions of the receptor is of prognostic relevance for the affected patients.

## Supporting Information

Figure S1 **PLA_2_R Construct.** *Panel A*: PLA_2_R isoform 1 (UniProtKB/Swiss-Prot: Q13018.2) was cloned into the pEGFP-N1 vector. *[EGFP, enhanced green fluorescent protein; SV40, simian vacuolating virus 40; Kan^R^/Neo^R^, kanamycin & neomycin resistance; HSV TK, herpes simplex virus type I thymidine kinase; CMV IE, cytomegalie virus immediate early protein 1; pUC, plasmid cloning vector created in the University of California; ori, origin of replication; PLA_2_R, phospholipase A_2_ receptor; f1 ori, phage-derived origin of replication; p, plasmid]. Panel B and C:* Reactivity of our construct was validated by Indirect Immunofluorescence (Panel B) and Western Blot (Panel C) using patient serum (iMGN = anti-PLA_2_R positive sample; Control = anti-PLA_2_R negative sample), anti-GFP and anti-PLA_2_R as primary antibody. Because of fusion with GFP (30 kDa), the recombinant fusion protein had an apparent molecular mass of 210 kDa.
(TIF)

Figure S2 **ALBIA (Luminex) IgG_4_ compared to ALBIA (Luminex) IgG.** *Panel A:* Again the median of ALBIA fluorescence units of anti-PLA_2_R positive samples were compared to samples negative for anti-PLA_2_R antibodies and control samples. Fluorescence values of anti-PLA_2_R positive samples were significantly higher than values of anti-PLA_2_R negative samples and of controls (p-values<0.0001). [Median with interquartile range]. *Panel B:* ALBIA readings of samples tested with IgG_4_ as secondary were also analyzed according to antibody titres determined on CB-IIF. Like the ALBIA IgG, samples with a high titre on CB-IIF had often a high fluorescent value on the bead-based assay but again ALBIA readings did not correlate with CB-IIF titres. [Whiskers: 2.5–97.5 percentile]. *Panel C:* This ROC curve compares the ALBIA assay using anti-human IgG_4_ versus anti-human IgG as secondary antibody. The difference between the two assays is marginal: AUC for anti-human IgG_4_ is 0.963 as opposed to 0.978 for anti-human IgG.
(TIF)

Figure S3 **Epitope mapping.** *Panel A: IgG (all subclasses) as secondary antibody.* Peptide membranes were tested with 10 randomly selected samples, 3 negative and 7 positive for anti-PLA_2_R antibodies as well as 5 normal healthy controls. High background signals were observed as well as some strong dots that varied depending on the sample and were therefore not considered as potential epitopes. *Panel B: IgG_4_ as secondary antibody.* Peptide membranes were also incubated with HRP conjugated anti-human IgG_4_ antibody (1∶1000; Jackson ImmunoResearch) as secondary. Besides healthy controls and patient serum samples, commercial rabbit anti-PLA_2_R (1∶500; Abcam; Immunogen: synthetic peptide derived from the C-terminal domain of human PLA_2_R) was also tested on the membrane. Seven potential epitopes (red boxes) were identified.
(TIF)

Figure S4 **ALBIA of synthesized PLA_2_R peptides.** For verifying potential epitopes, synthetic peptides (see Table 1) were tested by ELISA. Absorbance of patient samples tested positive on the CB-IIF assay was higher than of patient samples tested negative and normal healthy control samples but the difference was not statistically significant (p>0.05).
(TIF)

Table S1 **ALBIA units of sera tested on SPOT.** Samples were run in duplicates and values represent the mean value. [*NHC, normal healthy control].*
(DOCX)

Methods S1 **Protein Coupling, Blocking and Storage onto Microspheres.**
(DOCX)
