# Supplementary figures and images for: An Anti-Phospholipase A2 Receptor Quantitative Immunoassay and Epitope Analysis in Membranous Nephropathy Reveals Different Antigenic Domains of the Receptor
Source: PLoS One. 2013 Apr 29;8(4):e61669. doi: 10.1371/journal.pone.0061669 (PMC3639255; doi:10.1371/journal.pone.0061669)

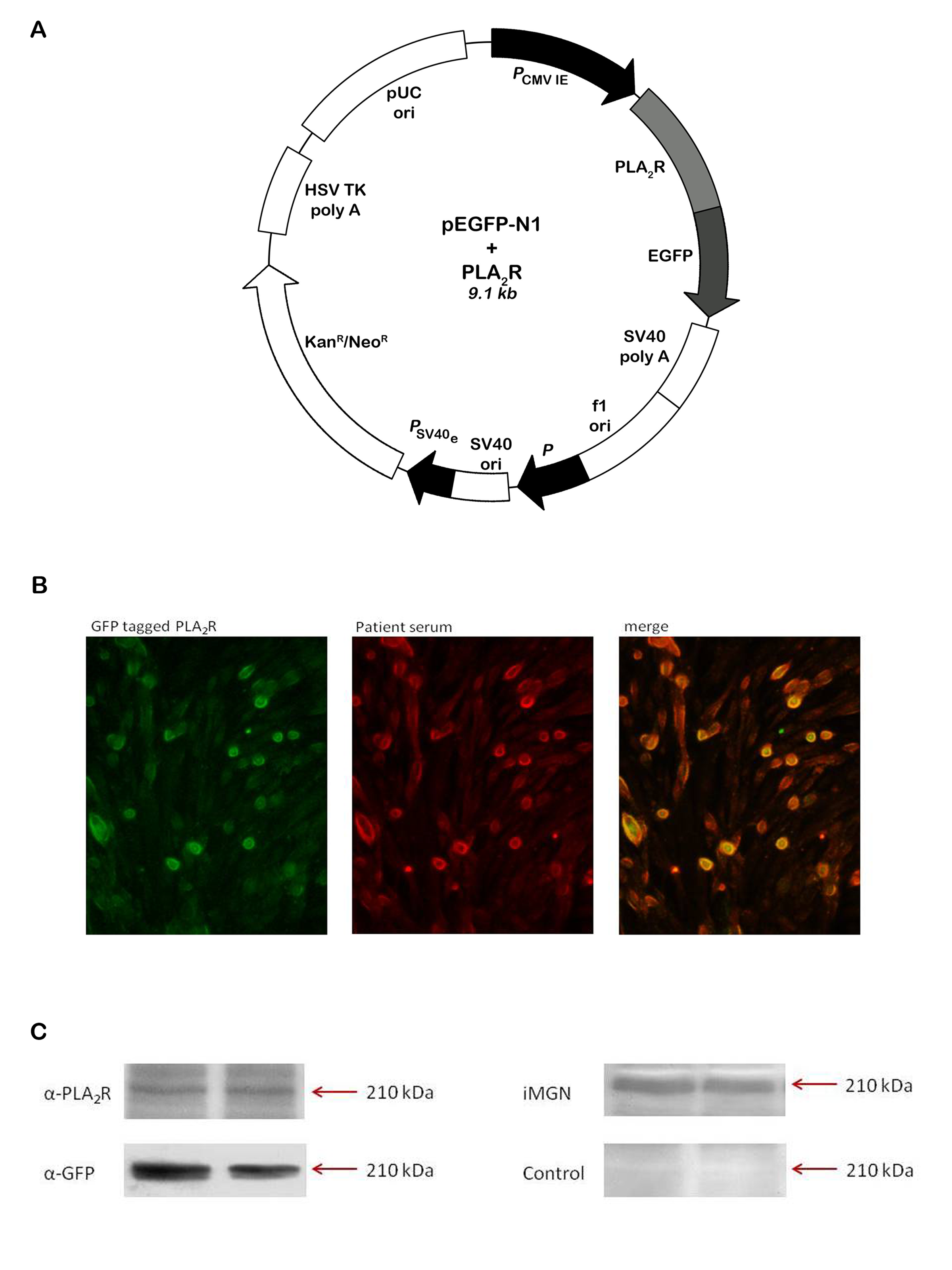

Supplement: Figure S1 — PLA2R Construct. Panel A: PLA2R isoform 1 (UniProtKB/Swiss-Prot: Q13018.2) was cloned into the pEGFP-N1 vector. [EGFP, enhanced green fluorescent protein; SV40, simian vacuolating virus 40; KanR/NeoR, kanamycin & neomycin resistance; HSV TK, herpes simplex virus type I thymidine kinase; CMV IE, cytomegalie virus immediate early protein 1; pUC, plasmid cloning vector created in the University of California; ori, origin of replication; PLA2R, phospholipase A2 receptor; f1 ori, phage-derived origin of replication; p, plasmid]. Panel B and C: Reactivity of our construct was validated by Indirect Immunofluorescence (Panel B) and Western Blot (Panel C) using patient serum (iMGN = anti-PLA2R positive sample; Control = anti-PLA2R negative sample), anti-GFP and anti-PLA2R as primary antibody. Because of fusion with GFP (30 kDa), the recombinant fusion protein had an apparent molecular mass of 210 kDa. (TIF) [file pone.0061669.s001.tif]

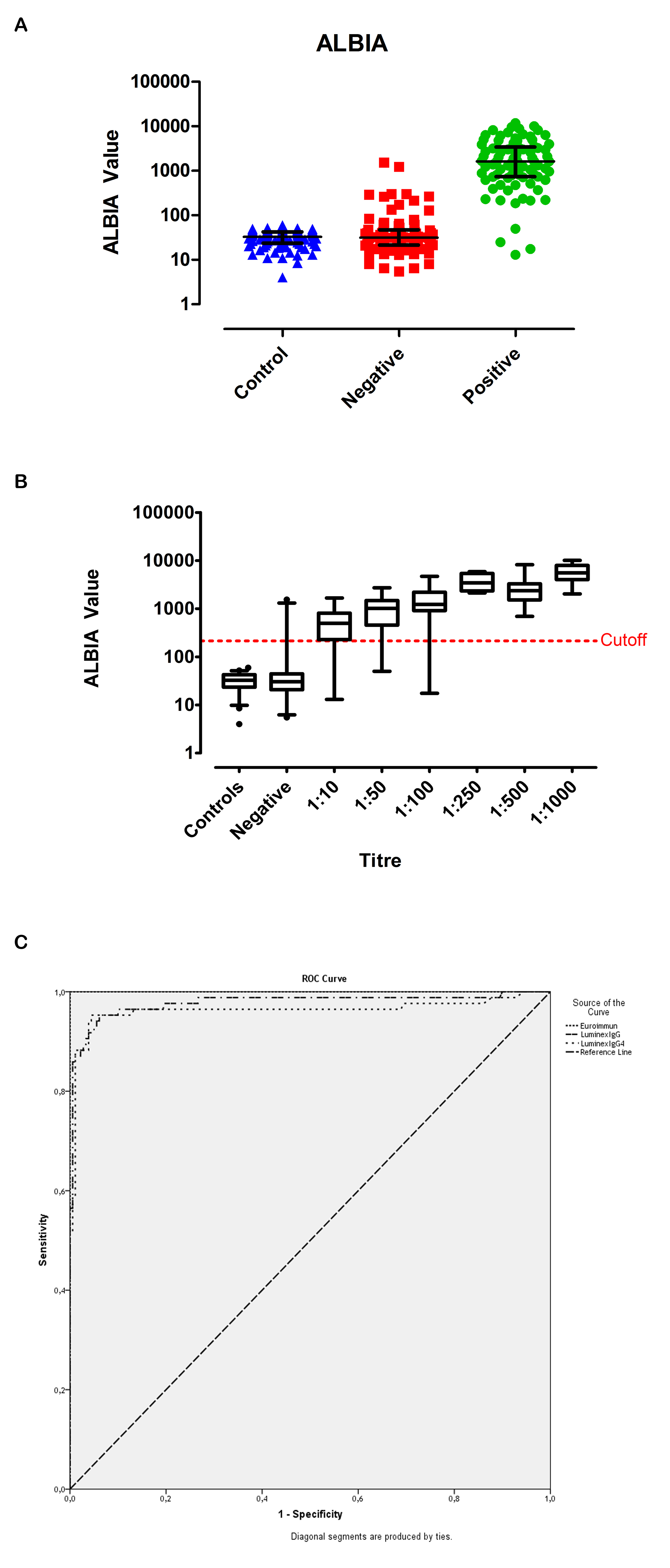

Supplement: Figure S2 — ALBIA (Luminex) IgG4 compared to ALBIA (Luminex) IgG. Panel A: Again the median of ALBIA fluorescence units of anti-PLA2R positive samples were compared to samples negative for anti-PLA2R antibodies and control samples. Fluorescence values of anti-PLA2R positive samples were significantly higher than values of anti-PLA2R negative samples and of controls (p-values<0.0001). [Median with interquartile range]. Panel B: ALBIA readings of samples tested with IgG4 as secondary were also analyzed according to antibody titres determined on CB-IIF. Like the ALBIA IgG, samples with a high titre on CB-IIF had often a high fluorescent value on the bead-based assay but again ALBIA readings did not correlate with CB-IIF titres. [Whiskers: 2.5–97.5 percentile]. Panel C: This ROC curve compares the ALBIA assay using anti-human IgG4 versus anti-human IgG as secondary antibody. The difference between the two assays is marginal: AUC for anti-human IgG4 is 0.963 as opposed to 0.978 for anti-human IgG. (TIF) [file pone.0061669.s002.tif]

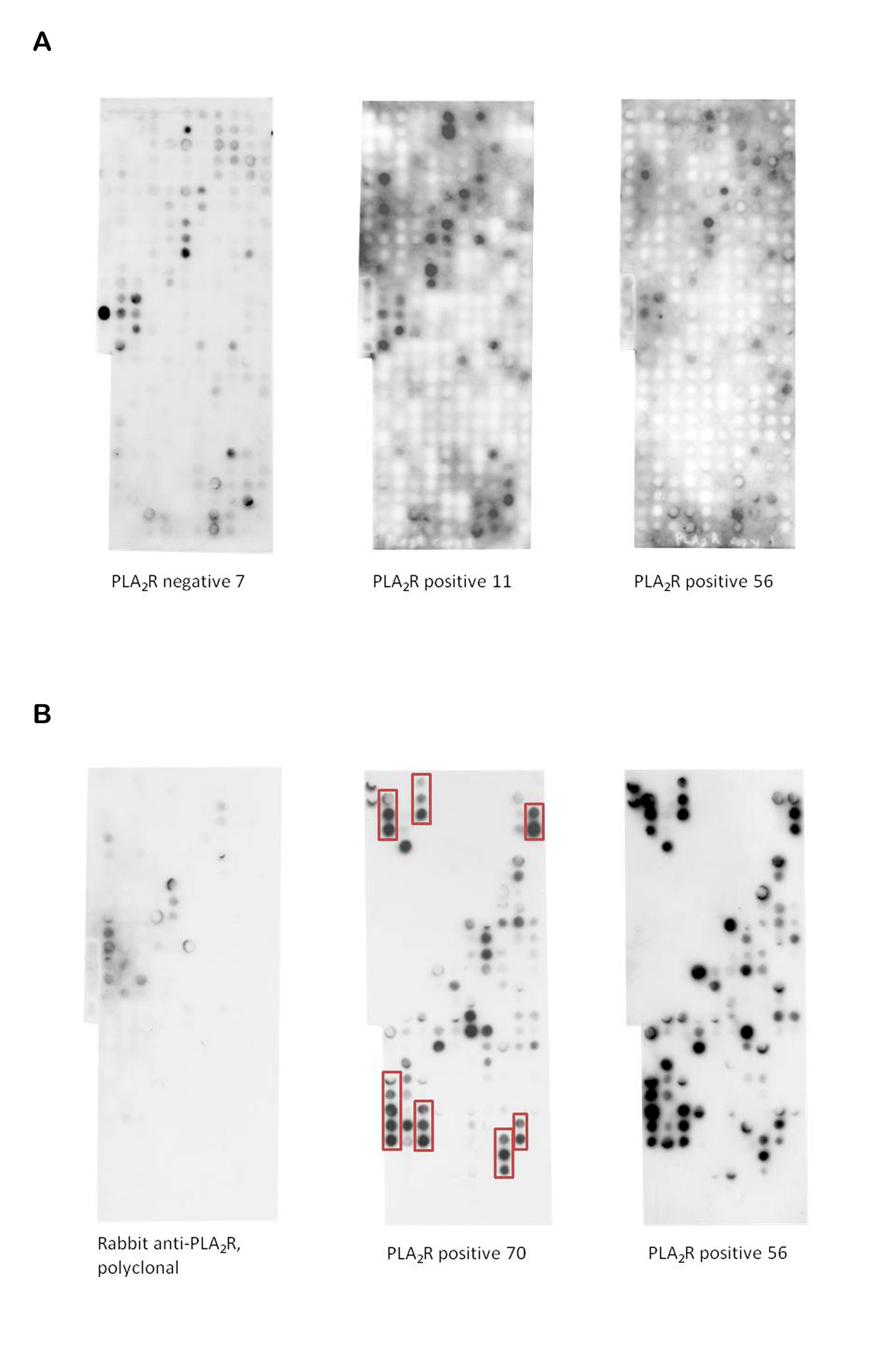

Supplement: Figure S3 — Epitope mapping. Panel A: IgG (all subclasses) as secondary antibody. Peptide membranes were tested with 10 randomly selected samples, 3 negative and 7 positive for anti-PLA2R antibodies as well as 5 normal healthy controls. High background signals were observed as well as some strong dots that varied depending on the sample and were therefore not considered as potential epitopes. Panel B: IgG4 as secondary antibody. Peptide membranes were also incubated with HRP conjugated anti-human IgG4 antibody (1∶1000; Jackson ImmunoResearch) as secondary. Besides healthy controls and patient serum samples, commercial rabbit anti-PLA2R (1∶500; Abcam; Immunogen: synthetic peptide derived from the C-terminal domain of human PLA2R) was also tested on the membrane. Seven potential epitopes (red boxes) were identified. (TIF) [file pone.0061669.s003.tif]

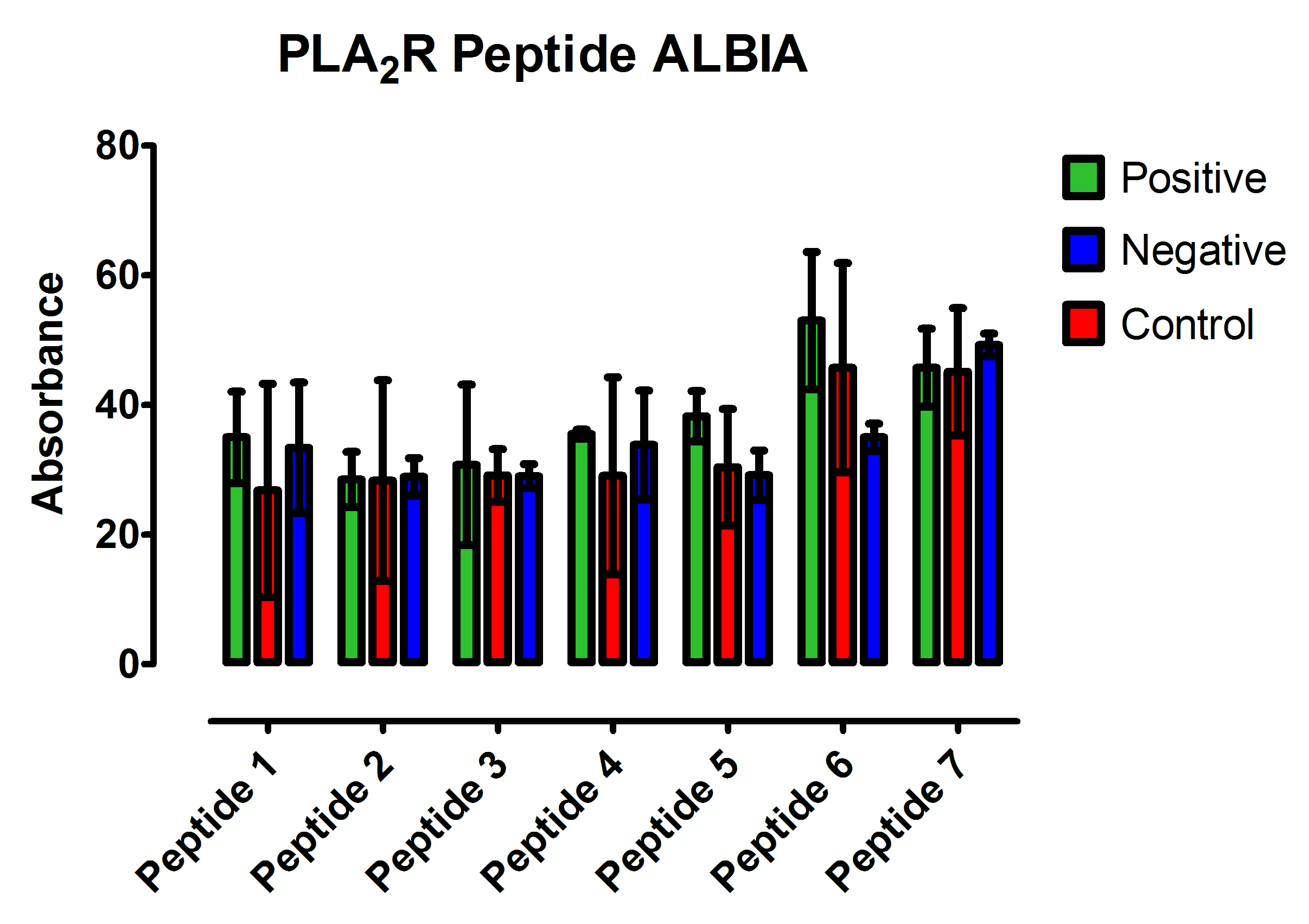

Supplement: Figure S4 — ALBIA of synthesized PLA2R peptides. For verifying potential epitopes, synthetic peptides (see Table 1) were tested by ELISA. Absorbance of patient samples tested positive on the CB-IIF assay was higher than of patient samples tested negative and normal healthy control samples but the difference was not statistically significant (p>0.05). (TIF) [file pone.0061669.s004.tif]
