# Supplementary material for: An Anti-Phospholipase A2 Receptor Quantitative Immunoassay and Epitope Analysis in Membranous Nephropathy Reveals Different Antigenic Domains of the Receptor
Source: PLoS One. 2013 Apr 29;8(4):e61669. doi: 10.1371/journal.pone.0061669 (PMC3639255; doi:10.1371/journal.pone.0061669)
Supplement: Table S1 — ALBIA units of sera tested on SPOT. Samples were run in duplicates and values represent the mean value. [NHC, normal healthy control]. (DOCX) [file pone.0061669.s005.docx]

| **Sample #** | **IIF-CBA** | **ALBIA IgG** | **ALBIA IgG_4_** |
| --- | --- | --- | --- |
| 11 | 1:500 | 6400 | 8218,5 |
| 12 | 1:250 | 8686,5 | 3885,5 |
| 48 | 1:500 | 4000 | 6625 |
| 56 | 1:100 | 3747 | 1942,5 |
| 70 | 1:100 | 8444 | 1433 |
| 74 | 1:1000 | 17904,5 | 8178,5 |
| 78 | 1:500 | 12565,5 | 2088,5 |
|  |  |  |  |
| 4 | negative | 61 | 8 |
| 5 | negative | 38,5 | 16 |
| 7 | negative | 29 | 29 |
|  |  |  |  |
| NHC (110) | negative | 90 | 45,5 |
| NHC (132) | negative | 91 | 29,5 |
| NHC (131) | negative | 216,5 | 39,5 |
| NHC (130) | negative | 174 | 39,5 |
| NHC (129) | negative | 107,5 | 51 |
