# Supplementary material for: An Anti-Phospholipase A2 Receptor Quantitative Immunoassay and Epitope Analysis in Membranous Nephropathy Reveals Different Antigenic Domains of the Receptor
Source: PLoS One. 2013 Apr 29;8(4):e61669. doi: 10.1371/journal.pone.0061669 (PMC3639255; doi:10.1371/journal.pone.0061669)
Supplement: Methods S1 — Protein Coupling, Blocking and Storage onto Microspheres. (DOCX) [file pone.0061669.s006.docx]

**Methods S1**

**Protein Coupling, Blocking and Storage onto Microspheres**

| Activation Buffer | Coupling Buffer | Wash Buffer | Blocking/Storage Buffer |
| --- | --- | --- | --- |
| 0,1 M NaH_2_PO_4_ | 0,14 M NaCl | PBS-T (0,05% Tween) | PBS |
| 5 M NaOH | 0,01 NaPO_4_ | pH ~ 7,2 | 1% BSA |
| pH ~ 6,2 | pH ~ 7,2 (PBS) |  | 0,05% NaN_3_ |

10 mg of EDC and NHS were dissolved in 200 *µ*l of Activation Buffer. A desired volume of beads (Luminex Corp., Austin, TX, USA) was pipetted into USA Scientific, Inc. Micro Centrifuge tubes and centrifuged at 14,000 rpm for 1 min. The supernatant was carefully decanted, the desired amount of activation buffer was added and the beads were resuspended by gentle sonication and vortexing. Diluted EDC and NHS were added and the beads sonicated and vortexed again followed by a 20 minute-incubation in the dark at room temperature.

| Starting Bead  Stock (*µ*l) | Activation Buffer  Volume (*µ*l) | EDC Solution (*µ*l) | NHS Solution (*µ*l) | Total Volume (*µ*l) |
| --- | --- | --- | --- | --- |
| 10 | 40 | 5 | 5 | 50 |
| 30-50 | 80 | 10 | 10 | 100 |
| 100 | 160 | 20 | 20 | 200 |

While the beads were incubating, protein samples were diluted to the optimal concentration in Coupling Buffer (usually 50 *µ*g/ml). After incubation, beads were centrifuged at 14,000 rpm for 3 minutes and the supernatant decanted before adding coupling buffer at 2-3 times the original bead volume. The microspheres were again sonicated and vortexed before centrifugation at 14,000 rpm for another 3 minutes.

| Starting Bead Volume(*µ*l) | Diluted Protein Volume (*µ*l) |
| --- | --- |
| 10 | 50 |
| 30 | 75 |
| 50 | 100 |
| 100 | 200 |

The supernatant was decanted and protein was coupled to microspheres by adding the optimal amount of protein to the microspheres, which were resuspended as described above. The beads were then incubated overnight at 4°C on rotator and then stored at 4^o^C in the dark until required for use.
